# AKI in a Patient on Etanercept for Juvenile Idiopathic Arthritis

**DOI:** 10.34067/KID.0000000000000336

**Published:** 2024-02-29

**Authors:** Riyam Al-Sammarraie, Innocent Segamwenge, Stuart Robertson

**Affiliations:** Betsi Cadwaladr University Health Board-Wrexham Maelor Hospital, Wrexham, United Kingdom

**Keywords:** AKI, cancer, immunology, immunosuppression

## Abstract

**Figure:**
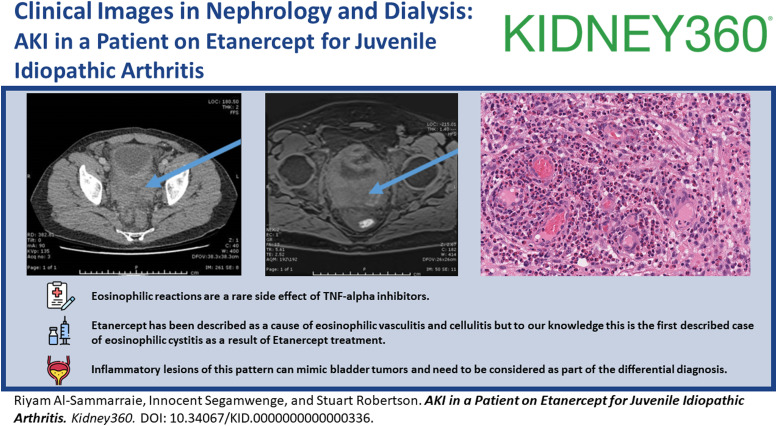

## Case Description

A 43-year-old man was admitted to the hospital with altered vision. He had a history of juvenile idiopathic arthritis, and he had been on etanercept (59 mg weekly) for 3 years before admission.

He was found to have bilateral papilledema; a computed tomography (CT) scan of his head revealed features of empty sella syndrome and thinning of the pituitary gland. Lumbar puncture revealed a cerebral spinal fluid opening pressure of 35 mm H_2_O, which required therapeutic drainage. It also had a white blood cell count of 3×10^6/L (no eosinophils) and red blood cell count 12×10^6/L. This was deemed to be idiopathic intracranial hypertension.

**Figure 1:**
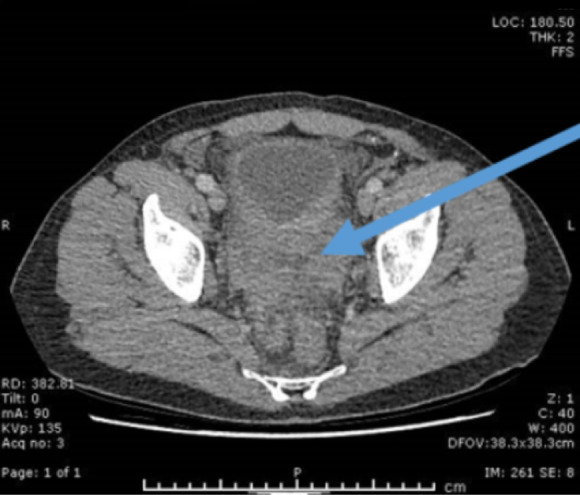
**Cross-sectional CT image showing abnormal bladder suspicious of either an inflammatory or malignant process.** CT, computed tomography.

**Figure 2:**
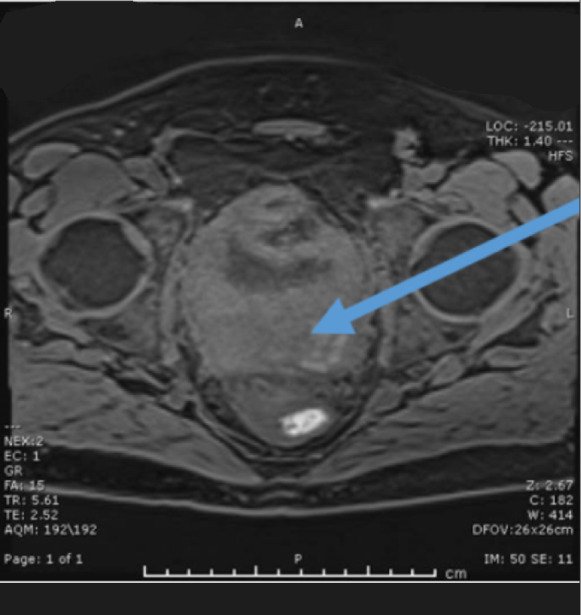
**Cross-sectional magnetic resonance imaging scan showing marked posterior bladder wall thickening.** Space in the upper left and right corners has been obscured for patient confidentiality purposes.

Blood tests revealed a marked AKI (serum creatinine: 5.5 mg/dl, eGFR: 11 ml/min per 1.73 m^2^) with evidence of bilateral hydronephrosis on ultrasound. Initial CT scan showed an abnormal bladder and retroperitoneal area suspicious for either a primary inflammatory or malignant process (Figure 1). The remainder of the chest, abdominal, and pelvic CT was normal. Pelvic and bladder magnetic resonance imaging showed an extensive urinary bladder mass suspicious of metastatic malignancy (Figure 2).

**Figure 3:**
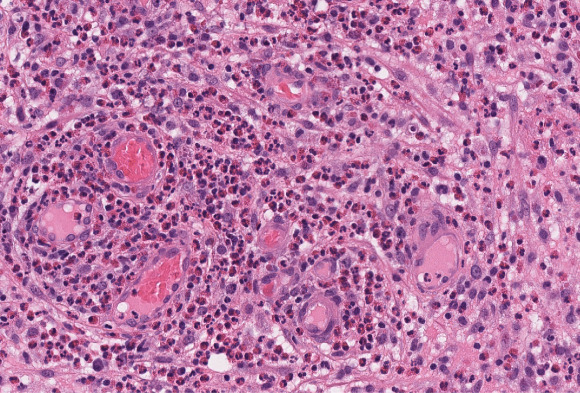
Histopathologic image of the bladder showing features of eosinophilic cystitis.

The patient initially had bilateral nephrostomies inserted, which allowed his kidney function to return to normal. He had an ongoing swinging fever and elevated inflammatory indices (C-reactive protein 200–300 mg/L, white blood cell: 14–22×10^0/L, neutrophils 11–20×10^9/L, eosinophils marginally high at 0.6×10^9/L). Biopsies were obtained from his bladder and prostate, which showed a marked eosinophilic inflammation (Figure 3).

After the biopsy, the patient was started on prednisolone 40 mg. This was followed by rapid resolution of C-reactive protein and fever. Bilateral nephrostograms showed free flow of urine into the bladder, which allowed nephrostomies to be removed. The eosinophil count recovered to normal.

Steroids were gradually weaned off over 1 year with no radiologic or biochemical recurrence. Acetazolamide was also subsequently stopped with no recurrence of headache or papilledema. The final diagnosis was etanercept-induced eosinophilic cystitis, prostatitis, and periaortitis.

## Discussion

Eosinophilic cystitis is a rare pathology characterized by inflammation of the bladder wall with infiltration of eosinophils. It has a range of presentations that are often misleading, sometimes mimicking bladder tumors, therefore, requiring a biopsy and histopathologic examination to rule out malignancy.^1^

A review of the literature revealed several previously described cases of different eosinophilic complications that were linked to TNF-*α* inhibitors; a few of them were specifically linked to etanercept.^2,3^

Etanercept is a biologic TNF-*α* inhibitor, acting as a soluble TNF receptor, which binds to TNF-*α* and blocks its effect.^4^ It is unclear how treatment with TNF-*α* inhibitors can lead to eosinophilia; it has been suggested that suppression of TNF-*α* leads to a shift of the Th1–Th2 balance, leading ultimately to eosinophilia.^5^

To the best of our knowledge, this is the first described case of this pattern of eosinophilic infiltration. This case and the previously published case studies that describe a possible etiologic link between eosinophilic syndromes and TNF-*α* inhibitors warrant further research into this link.

## Teaching Points

- Eosinophilic reactions are a rare side effect of TNF-*α* inhibitors.
- Etanercept has been described as a cause of eosinophilic vasculitis and cellulitis, but to our knowledge, this is the first described case of eosinophilic cystitis as a result of etanercept treatment.
- Inflammatory lesions of this pattern can mimic bladder tumors and need to be considered as part of the differential diagnosis.
